# α-Tocopherol protects keratinocytes against ultraviolet A irradiation by suppressing glutathione depletion, lipid peroxidation and reactive oxygen species generation

**DOI:** 10.3892/br.2014.236

**Published:** 2014-02-10

**Authors:** CHI-MING WU, YA-LI CHENG, YOU-HUA DAI, MEI-FEI CHEN, CHEE-CHAN WANG

**Affiliations:** Department of Cosmetic Science, Vanung University, Tao-Yuan 32061, Taiwan, R.O.C.

**Keywords:** α-tocopherol, keratinocytes, ultraviolet A radiation, glutathione, lipid peroxidation

## Abstract

This study aimed to investigate whether α-tocopherol is able to protect keratinocytes against ultraviolet A (UVA) radiation by increasing glutathione (γ-glutamylcysteinylglycine; GSH) levels or decreasing lipid peroxidation and reactive oxygen species (ROS) generation. The cell survival fraction was 43.6% when keratinocytes were irradiated with UVA at a dose of 8 J/cm^2^. α-Tocopherol was added prior to UVA irradiation and the cell viability was assayed. The cell survival fractions were 60.2, 77.1, 89.0, 92.9 and 96.2% when α-tocopherol was added at concentrations of 2.9, 5.9, 8.8, 11.8 and 14.7 IU/ml, respectively. These results suggested that α-tocopherol is capable of protecting keratinocytes against UVA irradiation. Furthermore, the levels of GSH, lipid peroxidation and ROS were measured. The levels of GSH were 0.354 and 0.600 mmol/g protein in keratinocytes irradiated with UVA (8 J/cm^2^) and in non-irradiated cells, respectively, whereas they were 0.364, 0.420, 0.525, 0.540 and 0.545 mmol/g protein when α-tocopherol was added at concentrations of 2.9, 5.9, 8.8, 11.8 and 14.7 IU/ml, respectively. The levels of lipid peroxidation were 20.401 or 5.328 μmol/g [malondialdehyde (MDA)/protein] in keratinocytes irradiated with UVA (8 J/cm^2^) and in non-irradiated cells, respectively, whereas they were 11.685, 6.544, 5.847, 4.390 and 2.164 μmol/g (MDA/protein) when α-tocopherol was added at concentrations of 2.9, 5.9, 8.8, 11.8 and 14.7 IU/ml, respectively. The levels of ROS were 3,952.17 or 111.87 1/mg protein in keratinocytes irradiated with UVA (8 J/cm^2^) and in non-irradiated cells, respectively, whereas they were 742.48, 579.36, 358.16, 285.63 and 199.82 1/mg protein when α-tocopherol was added at concentrations of 2.9, 5.9, 8.8, 11.8 and 14.7 IU/ml, respectively. These findings suggested that α-tocopherol protects keratinocytes against UVA irradiation, possibly through increasing the levels of GSH or decreasing the levels of lipid peroxidation and ROS generation.

## Introduction

Ultraviolet (UV) radiation (200–400 nm) is associated with a number of acute and chronic effects on the skin, which may result in inflammation, immunosuppression, premature aging and the development of malignancies (1). Ultraviolet A (UVA) radiation (320–400 nm), which is not absorbed by the ozone layer, comprises >95% of the UV light that reaches the surface of the earth. UVA penetrates the epidermis and affects the epidermal and dermal layers of the skin. At the cellular level, UVA exposure causes significant oxidative stress via generation of reactive oxygen species (ROS), such as singlet oxygen, hydroxyl radical, superoxide anion and hydrogen peroxide (2). ROS are rapidly removed by non-enzymatic antioxidants, particularly glutathione (γ-glutamylcysteinylglycine; GSH), as well as enzymatic antioxidants, such as catalase, superoxide dismutase, GSH peroxidase and GSH reductase, which maintain the pro-/antioxidant balance resulting in cell and tissue stabilization. However, a surplus of ROS may overwhelm the skin antioxidant defense mechanisms causing a pro-/antioxidant disequilibrium. The overproduction of ROS induces oxidation of nucleic acids, proteins and membrane lipids, which may result in intracellular GSH and NADH/NADPH depletion and, therefore, energy loss from the cell. UV-generated ROS also affects the regulation of the gene expression of signaling molecules/cascades, such as mitogen-activated protein kinases, interrelated inflammatory cytokines, nuclear factor-κB and activator protein-1 (3).

α-Tocopherol is the most active form of vitamin E, a fat-soluble vitamin that exists in 8 different forms in humans, and is a powerful biological antioxidant. α-Tocopherol is considered to be the major membrane-bound antioxidant employed by cells (4). α-Tocopherol scavenges acylperoxyl radicals and, thus, exerts a protective effect against cell membrane lipid peroxidation induced by UVA (5–8). Moreover, α-tocopherol and ascorbic acid work together in a cyclic process (9). During the antioxidant reaction, α-tocopherol is converted to an α-tocopherol radical by the donation of a labile hydrogen to a lipid or lipid peroxyl radical. The α-tocopherol radical is thus reduced to the original α-tocopherol form by ascorbic acid (9).

Among the cutaneous antioxidants, the tripeptide GSH, plays a pivotal role in protecting skin cells against oxidative damage by directly scavenging ROS or by acting as a coenzyme in GSH peroxidase- or GSH S-transferase-catalyzed reactions (10,11). It was demonstrated that GSH is also involved in DNA repair and cell apoptosis (12,13). Moreover, GSH participates in a number of biological processes, such as mitochondrial respiration, inflammatory response, signal transduction, regulation of gene expression and cell proliferation (14). GSH is able to regenerate the most essential antioxidants, vitamins C and E, back to their active forms and reduce the tocopherol radical of vitamin E directly or indirectly, via the reduction of semidehydroascorbate to ascorbate.

This study aimed to investigate the protective activity of α-tocopherol against UVA in an experimental model of HaCaT human keratinocytes. The effects of α-tocopherol on UVA-induced cellular oxidative stress, particularly on the intracellular levels of GSH, lipid peroxidation and ROS were investigated.

## Materials and methods

### Materials

The HaCaT human keratinocyte cell line was obtained from the Food Industry Research and Development Institute (Hsinchu, Taiwan). Dulbecco’s modified Eagle’s medium (DMEM), heat-inactivated fetal calf serum (FCS), penicillin-streptomycin and trypsin-EDTA solutions were purchased from Gibco-BRL (Carlsbad, CA, USA). Malondialdehyde (MDA) was purchased from Merck KGaA (Darmstadt, Germany). Sterile dimethylsulfoxide (DMSO), 3-(4,5-dimethylthiazol-2-yl)-2,5-diphenyltetrazolium bromide (MTT), 5,5′-dithiobis-(2-nitrobenzoic acid) (DTNB), thiobarbituric acid (TBA), dichlorodihydrofluorescein diacetate (H_2_DCFDA) and α-tocopherol were purchased from Sigma-Aldrich (St. Louis, MO, USA).

### Cell culture

The HaCaT cells were grown in DMEM supplemented with heat-inactivated FCS (10%, v/v), streptomycin (100 U/ml) and penicillin (0.1 mg/ml) in a humidified atmosphere of 5% CO_2_ at 37°C. The culture medium was changed 3 times per week. The cells were subcultured following trypsinization and seeded in 6-well plates at a density of 1×10^5^ cells/cm^2^.

### UVA irradiation and pretreatment with α-tocopherol

The keratinocytes were pretreated with α-tocopherol (2.9–14.7 IU/ml) at 37°C for 24 h, irradiated and incubated in serum-free medium at 37°C for an additional 24 h. Stock solutions of α-tocopherol were dissolved in DMSO, with a final concentration of DMSO in the medium of 1% (v/v). Irradiated and non-irradiated control cells were treated with serum-free medium. Prior to UVA irradiation, the cells were washed with phosphate-buffered saline (PBS) and covered with a layer of PBS. Dishes with keratinocytes were irradiated (8 J/cm^2^ UVA) on ice-cold plates to eliminate UVA thermal stimulation. Non-irradiated cells were treated similarly and were incubated in the dark. For the irradiation, a solar simulator system (Bio-Sun; Vilber Lourmat, Marne-la-Vallée, France) with a fixed wavelength of 365 nm was used.

### MTT assay

Cell viability was monitored following UVA irradiation and pretreatment with α-tocopherol. MTT was used to quantify the metabolically active living cells. Mitochondrial dehydrogenases metabolize MTT to a purple formazan dye, which is measured photometrically at 570 nm using a spectrophotometer (15).

### Intracellular GSH levels

Intracellular GSH was measured using a reaction with DTNB (16). The keratinocytes were rinsed with PBS, scraped into cooled perchloric acid (1%, v/v) and sonicated. The aliquots were frozen for protein determination using the Bradford assay. The suspension was centrifuged (13,000 rpm for 10 min at 4°C) and the supernatant was used to measure GSH in the reaction with the reaction mixture [800 mmol/l Tris-HCl, 20 mmol/l EDTA (pH 8.2) and 20 mg/ml DTNB)]. A dilution series of GSH (0.15–1 mM) were prepared as standard. The absorbance was measured on a microplate reader at 412 nm.

### Lipid peroxidation

The levels of lipid peroxidation were determined by measuring MDA in the cell extract. MDA is the end product of lipid peroxidation, which is induced by free radicals or activated oxygen species when cells are irradiated by UVA. The amount of MDA was measured using the TBA assay (17). TBA reacts with MDA, yielding red complexes, which absorb at 535 nm. A dilution series of MDA (0.1–10 μM) were prepared as standard. The cells were washed with cooled PBS, scraped into trichloroacetic acid (2.8%, w/v), sonicated and aliquots were used for protein determination with the Bradford assay. The suspension was mixed with TBA (1%, w/v) in a ratio of 2:1, heated for 30 min at 95°C and centrifuged (13,000 rpm for 10 min at 4°C). The amount of MDA was determined spectrophotometrically.

### ROS generation

The radical-scavenging efficacy of α-tocopherol in irradiated cells was monitored by the dichlorodihydrofluorescein assay. The polar, pre-fluorescent dichlorodihydrofluorescein diacetate (H_2_DCFDA) undergoes deacetylation by cytosolic esterases to form dichlorodihydrofluorescein, which reacts with ROS to produce fluorescein. The fluorescence is monitored at specific excitation/emission wavelengths of 488/525 nm. Following irradiation and pretreatment with α-tocopherol, the cells were incubated with H_2_DCFDA (5 nmol/l) for 15 min at 37°C. The cells were then washed with PBS, scraped into 2 ml of PBS and sonicated (18). The fluorescence was measured using a spectrophotometer (LS 50B; PerkinElmer, Waltham, MA, USA) and the protein concentration was determined with the Bradford assay.

### Statistical analysis

The means ± standard error of the mean were calculated from at least 3 repeated groups in all the experiments. Statistical significance between groups was determined with the Student’s t-test. P<0.05 was considered to indicate a statistically significant difference between two groups.

## Results

### Modulation of cell viability in UVA-irradiated cells by α-tocopherol

**Figure 1:**
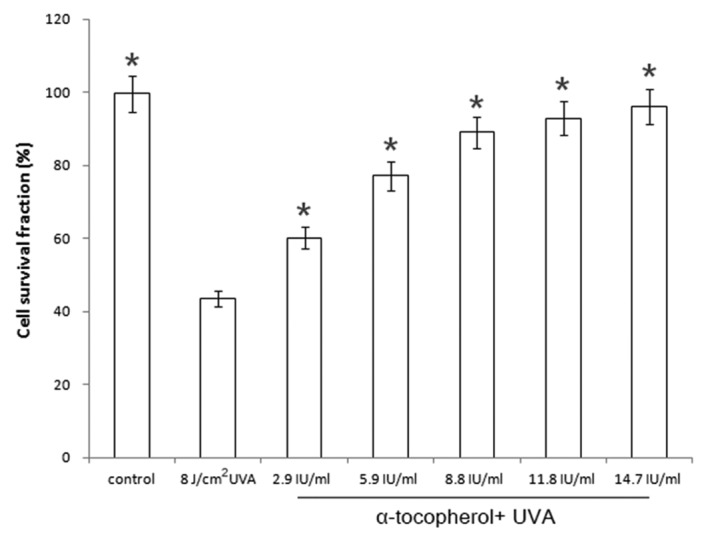
Modulation of cell viability in UVA-irradiated cells by α-tocopherol. The cell survival fractions were measured with the MTT assay when α-tocopherol was added at concentrations of 2.9, 5.9, 8.8, 11.8 and 14.7 IU/ml prior to UVA irradiation (8 J/cm^2^). Data are expressed as the means ± SD. ^*^P<0.05 compared to irradiated cells. UVA, ultraviolet A; SD, standard deviation.

The cell survival fraction was 43.6% when keratinocytes were irradiated with UVA at a dose of 8 J/cm^2^. The keratinocytes were pretreated with α-tocopherol (2.9–14.7 IU/ml) prior to UVA irradiation. The cell survival fractions were 60.2, 77.1, 89.0, 92.9 and 96.2% following addition of α-tocopherol at concentrations of 2.9, 5.9, 8.8, 11.8 and 14.7 IU/ml, respectively (Fig. 1). α-Tocopherol pretreatment suppressed the UVA-induced decrease in cell viability in a concentration-dependent manner. These findings suggest that α-tocopherol is capable of protecting keratinocytes against UVA irradiation.

### Prevention of UVA-induced GSH depletion by α-tocopherol

**Figure 2:**
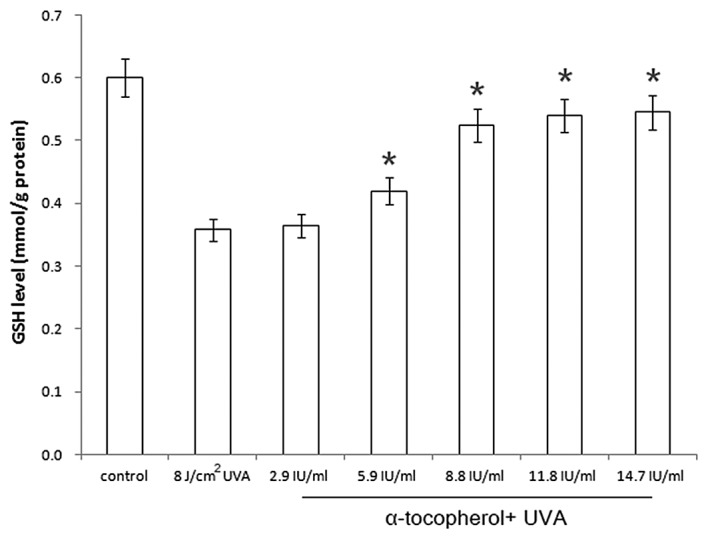
Prevention of UVA-induced GSH depletion by α-tocopherol. The keratinocytes were pretreated with α-tocopherol at concentrations of 2.9, 5.9, 8.8, 11.8 and 14.7 IU/ml and irradiated with UVA (8 J/cm^2^). The intracellular GSH levels were assayed. Data are expressed as the means ± SD. ^*^P<0.05 compared to irradiated cells. UVA, ultraviolet A; GSH, glutathione; SD, standard deviation.

As illustrated in Fig. 2, the GSH level in UVA-irradiated HaCaT cells (8 J/cm^2^) was decreased to ~50% of that in control cells (0.600→0.354 mmol/g protein). When α-tocopherol was added prior to UVA irradiation, the GSH levels in the cells were 0.364, 0.420, 0.525, 0.540 and 0.545 mmol/g protein at α-tocopherol concentrations of 2.9, 5.9, 8.8, 11.8 and 14.7 IU/ml, respectively. Therefore, the application of α-tocopherol to UVA-irradiated keratinocytes led to a dose-dependent prevention of GSH depletion.

### Modulation of UVA-induced lipid peroxidation by α-tocopherol

**Figure 3:**
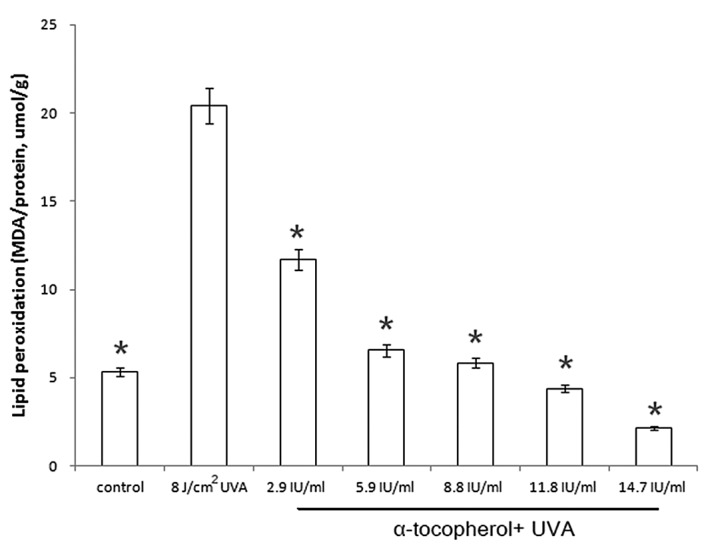
Modulation of UVA-induced lipid peroxidation by α-tocopherol. The keratinocytes were pretreated with α-tocopherol at concentrations of 2.9, 5.9, 8.8, 11.8 and 14.7 IU/ml and irradiated with UVA (8 J/cm^2^). The intracellular lipid peroxidation levels were assayed. Data are expressed as the means ± SD. ^*^P<0.05 compared to irradiated cells. UVA, ultraviolet A; MDA, malondialdehyde; SD, standard deviation.

The development of lipid peroxidation in irradiated HaCaT cells was measured using the TBA assay. Under our experimental conditions, there was a distinct increase in lipid peroxidation in the UVA-irradiated cells compared to the control cells (5.238→20.401 MDA/protein, μmol/g). As illustrated in Fig. 3, the amount of MDA was significantly reduced by α-tocopherol pretreatment.

### Modulation of UVA-induced ROS generation by α-tocopherol

**Figure 4:**
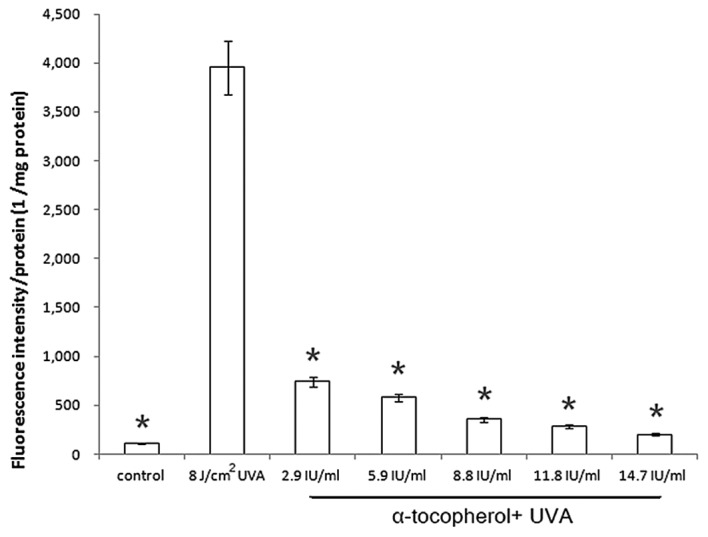
Modulation of UVA-induced ROS generation by α-tocopherol. The keratinocytes were pretreated with α-tocopherol at concentrations of 2.9, 5.9, 8.8, 11.8 and 14.7 IU/ml and irradiated with UVA (8 J/cm^2^). The intracellular ROS generation was assayed. Data are expressed as the means ± SD. ^*^P<0.05 compared to irradiated cells. UVA, ultraviolet A; ROS, reactive oxygen species; SD, standard deviation.

To investigate whether the addition of α-tocopherol affects UVA-induced ROS generation, a fluorescence assay using H_2_DCFDA was performed. α-Tocopherol significantly reduced ROS generation in UVA-irradiated keratinocytes in a dose-dependent manner (Fig. 4).

## Discussion

UV radiation is the principal factor causing skin cancer in humans. Several studies demonstrated that supplementation with antioxidants decreases UV-induced skin damage *in vitro* and *in vivo* (19). In this study, we demonstrated the ability of α-tocopherol to prevent and reduce UVA-related damage at the cellular level in human keratinocytes. Treatment of HaCaT cells with α-tocopherol prior to UVA exposure increased cell viability and suppressed intracellular GSH depletion, lipid peroxidation and ROS generation. The cell viability assay demonstrated that α-tocopherol protects HaCaT human keratinocytes against UVA-induced apoptosis. It is well known that during and following UVA irradiation, the generation of ROS is significantly increased in exposed cells (20,21). As UVA-related biological effects are primarily mediated by ROS, their elimination is essential for protection against UVA damage. The application of α-tocopherol led to a significant increase in cell survival in irradiated HaCaT cells. α-Tocopherol pretreatment exhibited maximal protection at the highest concentration tested.

Pretreatment of cells with α-tocopherol resulted in a concentration-dependent reduction in GSH depletion. The role of GSH in protecting the skin from oxidative damage caused by various chemicals and UV exposure has been well documented. Among non-enzymatic antioxidants, GSH is considered to be the most important, as it serves as a substrate for 2 major antioxidant enzymes, GSH-peroxidase and GSH-transferase, and it also participates in vitamin C and E regeneration (22). The GSH level is directly associated with the degree of lipid peroxidation in the cell membrane (23), as GSH participates in eliminating lipid peroxidation products, including 4-hydroxynonenal, by forming a GSH conjugate (24).

The cutaneous antioxidant system is complex and incompletely understood. We previously demonstrated that magnesium ascorbyl phosphate and coenzyme Q_10_ increased intracellular GSH levels (25). Kagan *et al* (26) reported that vitamin C is able to regenerate vitamin E from the α-tocopheroxyl radical. Furthermore, α-tocopherol and ascorbic acid work together in a cyclic process. During the antioxidant reaction, α-tocopherol is converted to an α-tocopherol radical through the donation of a labile hydrogen to a lipid or lipid peroxyl radical. The α-tocopherol radical is thus be reduced to the original α-tocopherol form by ascorbic acid (9). α-Lipoic acid was previously shown to elevate intracellular GSH levels *in vitro* by increasing *de novo* synthesis (27), an effect dependent upon the metabolic reduction of lipoic to dihydrolipoic acid. Dihydrolipoic acid is then released into the culture medium, where it reduces cystine to cysteine. Cysteine is readily taken up by the neutral amino acid transport system and utilized for GSH synthesis. Through this mechanism, lipoic acid enables cysteine to bypass the Xc^−^ transport system, which is weakly expressed in lymphocytes and inhibited by glutamate. Thereby, lipoic acid enables the key enzyme of GSH synthesis, γ-glutamylcysteine synthetase, which is regulated by an uptake-limited cysteine supply, to work at optimum conditions. However, the precise mechanism underlying the α-tocopherol-induced increase in intracellular GSH levels requires elucidation by further studies.

α-Tocopherol is the most active form of vitamin E in humans and is a powerful biological antioxidant, considered to be the major membrane-bound antioxidant employed by cells (4). The main antioxidant function of α-tocopherol is protection against lipid peroxidation (28). The overall process of lipid peroxidation includes three stages: initiation, propagation and termination. Once formed, peroxyl radicals are rearranged via a cyclization reaction to endoperoxides (precursors of MDA), with MDA as the final product (29). Our findings demonstrated that the amount of MDA was markedly reduced by treatment with α-tocopherol.

In conclusion, α-tocopherol protects keratinocytes against UVA irradiation, possibly through increasing the levels of GSH or decreasing the levels of lipid peroxidation and ROS generation.
